# Mapping the Binding between the Tetraspanin Molecule (Sjc23) of *Schistosoma japonicum* and Human Non-Immune IgG

**DOI:** 10.1371/journal.pone.0019112

**Published:** 2011-04-20

**Authors:** Chuang Wu, Pengfei Cai, Qiaocheng Chang, Lili Hao, Shuai Peng, Xiaojing Sun, Huijun Lu, Jigang Yin, Ning Jiang, Qijun Chen

**Affiliations:** 1 Key Laboratory of Zoonosis, The Ministry of Education, Jilin University, Changchun, China; 2 Laboratory of Parasitology, Institute of Pathogen Biology/Institute of Basic Medicine, Chinese Academy of Medical Sciences and Peking Union Medical College, Beijing, China; 3 College of Life Science and Technology, Southwest University of Nationalities, Chengdu, China; The George Washington University Medical Center, United States of America

## Abstract

**Background:**

Schistosomal parasites can establish parasitization in a human host for decades; evasion of host immunorecognition including surface masking by acquisition of host serum components is one of the strategies explored by the parasites. Parasite molecules anchored on the membrane are the main elements in the interaction. Sjc23, a member of the tetraspanin (TSP) family of *Schistosoma japonicum*, was previously found to be highly immunogenic and regarded as a vaccine candidate against schistosomiasis. However, studies indicated that immunization with Sjc23 generated rapid antibody responses which were less protective than that with other antigens. The biological function of this membrane-anchored molecule has not been defined after decades of vaccination studies.

**Methodology and Principal Findings:**

In this study, we explored affinity pull-down and peptide competition assays to investigate the potential binding between Sjc23 molecule and human non-immune IgG. We determined that Sjc23 could bind human non-immune IgG and the binding was through the interaction of the large extra-cellular domain (LED) of Sjc23 (named Sjc23-LED) with the Fc domain of human IgG. Sjc23 had no affinity to other immunoglobulin types. Affinity precipitation (pull-down assay) in the presence of overlapping peptides further pinpointed to a 9-amino acid motif within Sjc23-LED that mediated the binding to human IgG.

**Conclusion and Significance:**

*S. japonicum* parasites cloak themselves through interaction with human non-immune IgG, and a member of the tetraspanin family, Sjc23, mediated the acquisition of human IgG via the interaction of a motif of 9 amino acids with the Fc domain of the IgG molecule. The consequence of this interaction will likely benefit parasitism of *S. japonicum* by evasion of host immune recognition or immunoresponses. This is the first report that an epitope of schistosomal ligand and its immunoglobulin receptor are defined, which provides further evidence of immune evasion strategy adopted by *S. japonicum*.

## Introduction

Schistosomiasis is one of the most serious parasitic diseases in morbidity and mortality, which infects at least 207 million people and a large number of animals in 76 countries,with an estimated 700 million people at risk (World health Organization, February 2010). Schistosomiasis japonica, which is caused by the parasitization of adult male and female worms of *Schistosoma japonicum* within mesenteric or vesicular veins of the host, is the only zoonotic schistosomiasis that has proved to be the most difficult to be controlled among the 5 schistosome species that infect humans [1]–[3]. The parasites can thrive in a human host for decades. Vaccines based on the membrane components (or associated membrane proteins) have been extensively studied but with little success [4], [5]. It has been well-known that schistosomal parasites evade host immune expulsion through surface masking, molecular mimicking, and active modulation on host immune responses [6]. A variety of host molecules such as immunoglobulins, major histocompatibility complex products, complement components, blood group antigens have been found on the surface of the parasites inside the host [6], [7]. Acquisition of host components on the parasite surface was believed to benefit parasite by prevention of host recognition and immune attack [6]. So far, the non-filamentous paramyosin in association with parasite membrane of both *S. mansoni* and *S. japonicum* was the only molecule characterized as the receptor for un-specific binding of host (human and rodents) IgG and complement components, while the other parasite ligands that interact with host factors remain unidentified [7]–[10]. Though it has been hypothesized that the adherence of host serum factors on the surface could not only block recognition of anti-parasite antibodies, but also inhibit complement activity, it is, however, also possible that the parasites can actively influence host immune responses through interaction with immunoglobulins. While the surface location of the paramyosin is still a matter of debate [8], [10], [11], the tetraspanin (TSP) family proteins were also localized to the surface of both *S. mansoni* and *S. japonicum* [12]–[17]. The TSP family encoded in each schistosome genome contains more than 29 members and the expression profiles of various TSP members are quite diverse, some are with stage-specificity [15], [16], while others are universally expressed, indicating that they perform different functions during parasite development.

We have previously found that one member of the TSP family of *S. japonicum*, Sjc23, was more immunogenic than any other TSP members [14]. Sjc23 was expressed on the surface of de-tailed cercariae, lung-stage schistosomula, and adult worms [14]–[16]. Antibodies to Sjc23 were readily detected in the sera of patients of schistosomiasis and experimentally infected mice [14]. Further, it was found that antibody responses to Sjc23 were more rapid than to other schistosomal tegument antigens in mice either infected by the parasites or after immunization [14]. Intriguingly, the antibodies induced by Sjc23 were dominantly IgG2a type which has been proved to be inefficient in complement fixation and with less cytophilic property in ADCC (Antibody-dependent cell-mediated cytotoxicity). Here, we further explored the function of Sjc23 and found that it is a molecule with affinity to the Fc domain of human non-immune IgG. The binding was mapped to a 9 amino acid region in the loop of the large extracellular domain of Sjc23 (named Sjc23-LED). The findings further help us to better understand the immune evasion mechanism of *S. japonicum* and rational design of vaccines based on membrane proteins such as Sjc23.

## Results and Discussion

### Detection of Sjc23 expression on the surface of the parasites

**Figure 1:**
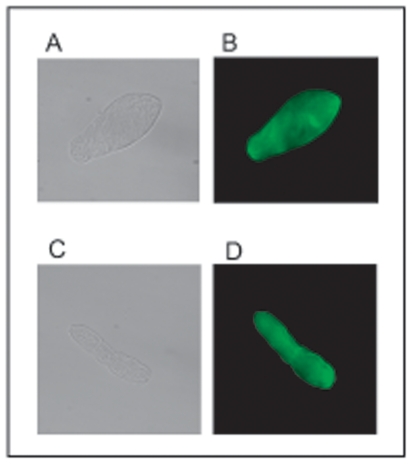
Detection of Sjc23 on the surface of *S. japonicun* with IFA. **A** and **C** are phase contrast images of cercarie and schistosomulum. B and D are the same parasites stained with specific anti-Sjc23 antibodies. The tail of the cercarie de-attached during washing steps.

In our earlier study [14], we showed that Sjc23 gene was actively transcribed in cercarie, schistosomulum, adult worm and egg stages and Sjc23 protein was detected in the parasite with Western-blot using antibodies specific to the Sjc23-LED. Here we used the same antibody to localise the protein on the surface of cercarie, schistosomulum and adult stage parasites (Fig. 1 and data not shown). Thus Sjc23 is a surface molecule as other tetraspanin family members.

### Cloning and expression of recombinant proteins

**Figure 2:**
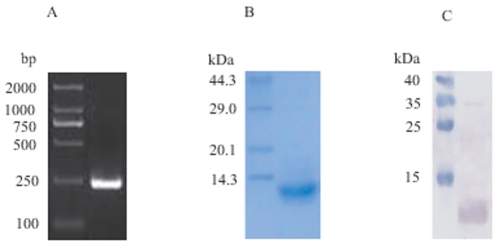
Cloning and expression of the large extracellular domain of Sjc23 (Sjc23-LED). **A** PCR product of the gene fragment coding for Sjc23-LED. The length of the fragment is 228 bp. **B** Purified His-tagged recombinant Sjc23-LED. The molecular weight of the His-Sjc23-LED is 12.4 kDa. **C** Western-blot confirmation of the recombinant protein with an anti-His-tag mAb.

In our previous investigations, we observed that the background recognition of the recombinant Sjc23-GST protein by sera from healthy humans were unexpectedly high. To further investigate the possible un-specific binding between the individual protein with human immunoglobulins, recombinant proteins of Sjc23-LED, GST and TSP-2 of *S. japonicum* were generated. The gene fragment encoding Sjc23-LED was amplified by PCR (Fig. 2A) and cloned into the pET-22b expression vector. The His-tagged recombinant Sjc23-LED protein was expressed and purified by a His GraviTrap column (GE Biosciences, Uppsala, Sweden). The molecular mass of the recombinant Sjc23-LED was 12.4 kDa (Fig. 2B). The expressed protein was confirmed by Western-blot using a mAb specific to the His-tag (Fig. 2C). Recombinant GST and TSP-2 were generated as described [14], [16].

### Sjc23-LED specifically bound human non-immune IgG

**Figure 3:**
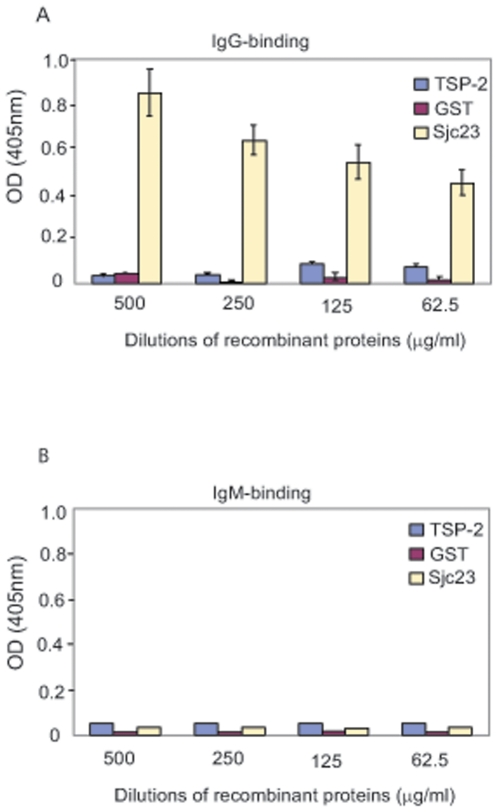
Binding of Sjc23-LED with human non-immune immunoglobulins in ELISA assay. Purified human plasma IgG and IgM (5 µg/ml) from individuals without background of schistosomiasis were coated on ELISA plates and the affinity of Sjc23, TSP2 and GST to human IgG (A) and IgM (B) was examined. Only Sjc23-LED binds to human IgG with concentration dependency. No adhesion to human IgM and other types of immunoglobulins (data not shown) was observed with any of the antigen.

To test the possible immunoglobulin binding property of the molecules generated above, a classical ELISA assay was performed. The three proteins, Sjc23-LED, GST and TSP-2, were incubated respectively with purified human IgG, IgM, IgA (Sigma, CA, USA) and IgE (Abcam, Cambridge, UK). Only Sjc23-LED was found to bind non-immune human IgG, while GST and TSP-2 did not show any binding activity (Fig. 3A). Further Sjc23-LED only bound human IgG, but not other types of immunoglobulins and albumin (Fig. 3B, Fig. S1). This explained our earlier observation that Sjc23-LED reacted with normal human sera in ELISA assays. Thus, Sjc23 is likely another schistosomal molecule, apart from paramyosin [7]–[10], with immunoglobulin-binding property.

**Figure 4:**
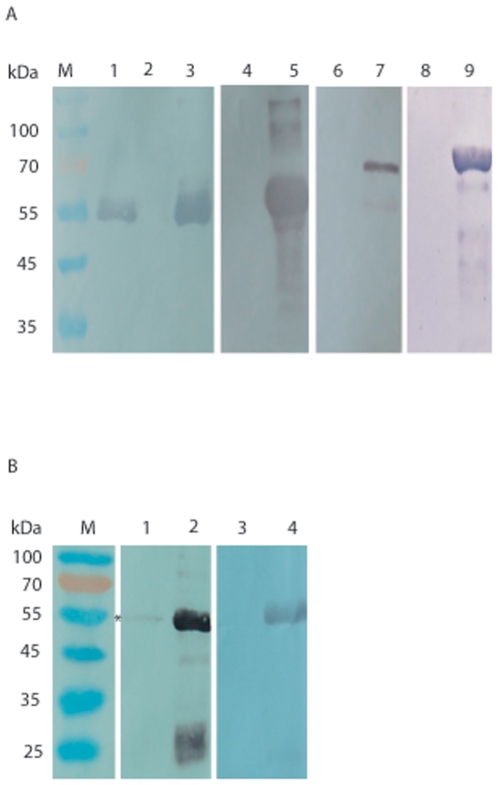
Binding of Sjc23-LED with immunoglobulins in pull-down assay. **A** Human IgG (lane 1), IgA (lane 4), IgE (lane 6), and IgM (lane 8) were incubated with Sjc23-LED bound Sepharose resin, after extensive washing, the proteins were resolved in SDS-PAGE and blotted to nylon film. The immunoglobulins that bound to Sjc23-LED was detected by using mAbs specific for human antibodies (γ-chain, α-chain,ε-chain or µ-chain specific). Only human IgG could specifically bind to Sjc23-LED, whereas the other antibody types did not. GST did not bind human IgG (lane 2). Lanes 3, 5, 7 and 9 were corresponding antibodies as controls for detection. **B** Porcine and bovine IgG was respectively incubated with Sjc23-LED bound Sepharose resin and only porcine IgG was marginally precipitated (lane 1, the band was indicated with an asterisk). Lane 2 and 4 were controls of the corresponding IgGs.

In order to confirm the binding between Sjc23-LED and human IgG, a pull-down assay was performed. We used Sjc23-LED as a bait protein immobilized on the nickel-Sepharose beads to capture the immunoglobulins that would interact with it. As shown in Fig. 4, only IgG was precipitated by Sjc23-LED immobilized Sepharose (Fig. 4A, lane 1), but not IgA, IgE or IgM. Pull-down assays with porcine and bovine IgG were also performed; however, very weak signal was observed with porcine IgG (Fig. 4B, lane 1), but no signal was detected with bovine IgG was seen (Fig. 4B, lane 3), indicating that Sjc23-LED mainly adhered human IgG. Compared with ELISA assay, pull-down assays require higher affinity between the ligand and receptor due to the high stringency of particle precipitation and washing steps. Thus the binding between Sjc23-LED and IgG was specific. Further, *S. japonicum* can establish infection in domestic pigs, though the parasitism and pathology in large stocks have not been well established when comparing to that in human, it is likely that the parasite is less adapted in animals than in humans. By comparing the binding property with immunoglobulins of different host origins, the adaptation of the parasites in different hosts may be further investigated. Nevertheless, combining the results of ELISA assays, we can conclude that the Sjc23 molecule of *S. japonicum* is a ligand for human non-immune IgG.

### Sjc23-LED bound the Fc domain of human IgG

**Figure 5:**
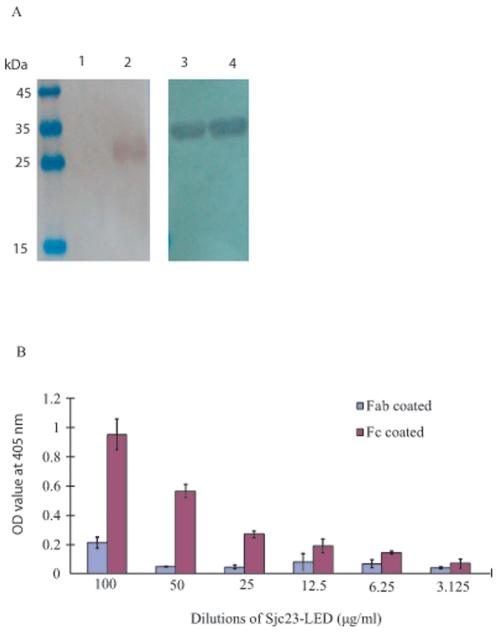
Binding of human IgG domains to Sjc23-LED. **A** In the pull-down assay, Fab and Fc fragments of human IgG were incubated with Sjc23-LED conjugated particles (lane 1 and 3) respectively. After washing, the binding was examined by anti-Fab and anti-Fc antibodies. One Fc fragment bound to Sjc23-LED (lane 3). No binding of Fab fragment to Sjc23-LED was detected. Lane 2 and 4 were loading control of Fab and Fc fragment respectively. **B** In ELISA assay, the plate was coated with Fab and Fc fragment respectively, and recombinant Sjc23-LED diluted from 100 to 3.125 µg/ml. Sjc23-LED only bound to Fc fragment and the binding was dilution-dependent.

To identify which region in human IgG that binds to Sjc23-LED, Fab and Fc fragments of human IgG (Merck, NJ, USA) were respectively incubated with Sjc23-LED immobilized Sepharose beads, and only the Fc fragment was precipitated (Fig. 5A lane 3), but not the Fab fragment. Further, the binding between Fc fragment and Sjc23-LED was also confirmed in ELISA assay (Fig. 5B). Thus, Sjc23 of *S. japonicum* is another molecule in schistosomal parasites, similar to paramyosin in binding with human non-immune IgG. An obvious question after this finding is the necessity of having two molecules exposed on the surface of the parasite to bind the same Fc domain of human IgG. One explanation could be that Sjc23 and paramyosin are differentially exposed on the parasite surface. It was reported that paramyosin was only exposed on the surface of schistosomula [18], while it was mainly located under the tegument membrane in other stages. Earlier studies indicated that Sjc23 was expressed in all parasite stages in the host [12]–[14]; however it could not be ruled out that the protein might be less exposed at certain stage, i.e. Sjc23 and paramyosin were differentially expressed during parasite development in the mammalian hosts. But the rapid immunoresponses in the host after infection strongly indicated the Sjc23 was exposed to the immune system from initial invasion [14]. Thus, it is likely that Sjc23 and paramyosin fulfilled different biological functions.

### A region of 9 amino acids in Sjc23-LED mediated IgG binding

**Figure 6:**
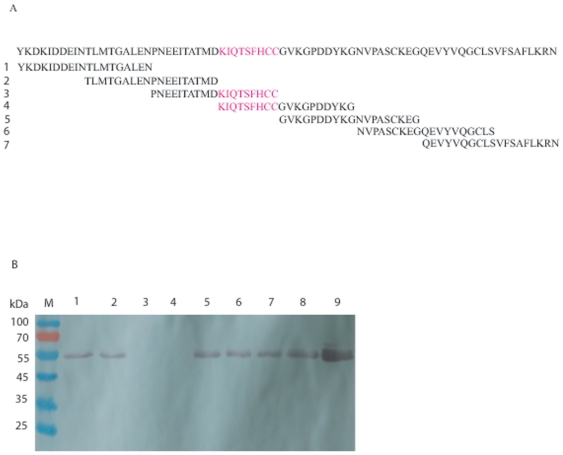
Defining the region of Sjc23-LED that bound human IgG. **A** Sequences of the synthetic peptides with overlapping regions. The amino acid sequence of Sjc23-LED was at the top and the 7 partially overlapping peptides were listed below. The sequence region with potential binding activity to human IgG was marked with pink color. **B** Inhibition of human IgG with Sjc23-LED by peptides. Sjc23-LED was incubated with human IgG in the presence of different peptides. Only peptide 3 and 4 showed inhibitory activity. Lane 8 and 9 were no peptide and loading control respectively.

**Figure 7:**
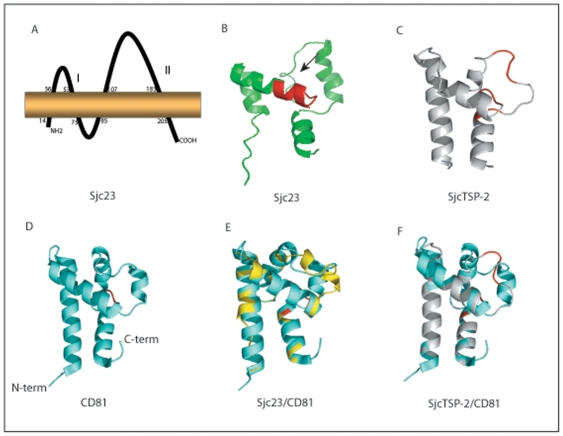
Structural modeling of Sjc23 and SjcTSP-2 molecule. **A** Schematic illustration of Sjc23 molecule anchoring in the membrane. Sjc23 molecule is a member of the tetraspanin family with 4 transmembrane regions and three loops. Loop I (aa 35–53) and II (107–183) are extracellular, while loop III is introcellular. **B** The structural modeling of the large extracellular domain II of Sjc23. This domain has a high similarity (>94% confidence) to the large extracellular domain of human CD81, another member of tetraspanin family. It is consisted of 4 helix regions and 4 low complex regions. The 9 amino acids region that mediated IgG binding was in helix 2 (red color and arrow-headed). **C**. Structure prediction of a SjcTSP-2 variant. The molecule has more low complex regions compared to other tetraspanins. Helix 2 is shorter than that of Sjc23. D. Structure of the large extracellular domain II of human CD81. E and F are overlapping of Sjc23 and SjcTSP-2 with human CD81.

The relatively small molecular mass of the Sjc23-LED made it possible to perform inhibition/competition assay with synthetic overlapping peptides against the binding of Sjc23-LED with IgG. Seven peptides were chemically synthesized with 9–10 amino acids overlapped between adjacent peptides. In the competition assays, peptide number 3 and 4 completely blocked the binding between Sjc23-LED and human IgG (Fig. 6A and B), but not the other peptides. There is a region of amino acids (-KIQTSFHCC-) that overlapped between the two peptides, thus it is likely the structure formed by the 9 amino acids mediated the binding of Sjc23-LED with the Fc fragment of human IgG. Structural analysis of the Sjc23-LED shows that the IgG-binding motif is located on the second helix of the molecule (Fig. 7). Interestingly, the -CCG- motif which is conserved in all TSP family proteins is located at the C-terminal side of the identified IgG-binding motif. TSP family proteins have been known to be promiscuous. The finding here may explain the mechanism of Ig-binding phenomenon in other organism [19]. However, due to the fact that TSP-2 of *S. japonicum* did not show any binding activity to Ig, it is most likely that the binding is structure-dependent. The 6 amino acids before the –CCG- motif in Sjc23-LED are very different from the sequences of other TSP proteins of schistosomal parasites (also see Fig. 7). Nevertheless, this is the first report that a defined motif of a schistosomal tegument protein with immunoglobulin-binding property has been mapped. Earlier study on paramyosin with fragmented recombinant proteins indicted that the binding region to a complement element (C9) was located closed to the C-terminal region of 122 amino acids, but the binding epitopes for both complement and IgG in the molecule have not been defined [9].

### The Sjc23 has a typical structure of tetraspanin family

Sequence scanning for hydrophobicity and potential transmembrane domains in the Sjc23 sequence, we found that, like other TSP members, Sjc23 is a molecule with 2 transmembrane domains (TM), a small and a large extracellular domain (EC1 and EC2, here called Sjc23-LED) and a small intracellular domain (Fig. 7A). We analyzed the tertiary structure of Sjc23-LED by blasting the sequence against the protein structure database (Protein Data Bank, PDB). The best hit was the structure of human CD81 which was another member of the TSP family [20] (Fig. 7). Further, Circular dichroism (CD) spectroscopy analysis of the recombinant Sjc23 protein indicated that the molecule was mainly composed of helical structures. The differences between Sjc23-LED and CD81 were mainly located in the low complex regions. In Sjc32-LED, the low complex regions were composed of more amino acids than other TSP members of the parasite (data not shown), this may indicate that the molecule is under pressure from the host, given the fact that both T and B cell epitopes were identified in the region of Sm23 [21].

Finally, the Sjc23 was the first tetraspanin identified in *S. japonicum* 20 years ago [22], [23] and it, as well as its homologous Sm23 from *S. mansoni*, has been regarded as a vaccine candidate which has been tested in several immunization and challenge trials in animals [24]–[28]. With the discovery of this study, it may be necessary to reconsider the vaccine development approach based on this antigen. To be able to generate high affinity antibodies that can overcome the unspecific Ig-binding, i.e. to compete out the masking immunoglobulins, might be a prerequisite for a successful development of the vaccine based on this antigen.

### Conclusion

In this study, we investigated the immunoglobulin binding property of the Sjc23 molecule, a member of the TSP family, which has been regarded as a vaccine candidate for schistosomiasis. It was found that the large extracellular domain of Sjc23, named Sjc23-LED, bind to human non-immune IgG via the interaction between a motif of 9 amino acids in the molecule and the Fc fragment of human IgG. This is the first report that the interaction of a schistosomal ligand and its human host receptor has been defined.

## Methods

### Detection of Sjc23 expression in the parasite by immunofluorescence

Detections of Scj23 protein in cercariae, schistosomula, adult worms were carried out with immunofluorescent assay using anti-Sjc23 antibodies raised in mice according to standard protocol. Briefly, parasites were fixed for 5 min in ice-cold acetone followed by blocking with 10% BSA in PBS for 2 h at room temperature. Primary antibodies to the large extracellular domain (LED) were incubated with the parasite at 4°C overnight. After washing 4 times in PBS buffer, the parasites were further incubated with a FITC-conjugated goat anti-mouse antibody for 2 h at room temperature. Parasites incubated with irrelevant sera and with the secondary antibodies alone were included as controls. The fluorescence was visualized with a Nikon fluorescence microscope (Japan). The expression of Sjc23 in different parasite development stages was further confirmed by Western blot with Sjc23 specific antibodies.

### Cloning of the gene fragment encoding the extra-cellular region of Sjc23 antigen (Sjc23-LED)

DNA was extracted from adult *S. japonicum*. PCR primers were designed based on the gene sequence of Sjc23 (GenBank accession number: M63706.1). The forward primer and reverse primer were 5′-GGATCCGTACAAGGATAAAATCGATG-3′ and 5′-CTCGAGGTTGCGTTTTAAGAATGCACTAAAG-3′, which carried a *Bam*H I and an *Xho* I restriction site respectively. PCR was performed under the following conditions: 4 min at 94°C for full denaturation,1 min at 94°C, 1 min at 58°C, 45 s at 72°C. The amplification was run for 32 circles with a last extension at 72°C for 10 min. The PCR product was analyzed by electrophoresis in a 1.5% agarose gel and visualized by staining the gel with ethidium bromide. The amplified product was gel purified using DNA Gel Extraction Kit (AxyGen, CA, USA) and cloned into the pET-22b expression vector. The recombinant plasmid named pET-22b-Sjc23-LED was verified by restriction enzyme digestion and sequencing.

### Expression, purification and verification of recombinant proteins

For protein expression,competent cells of the *E. coli* strain BL21(DE3) were transformed with pET-22b-Sjc23-LED plasmid and cultured in LB medium containing Ampicillin (100 µg/ml) at 37°C. Expression was initiated by addition of Isopropyl β-D-1-thiogalactopyranoside (IPTG) to a final concentration of 0.1 mM after the bacteria density reached 0.6–0.8 at OD_600._ The culture was incubated for 7 h at 25°C. Bacteria were harvested by centrifugation and the recombinant His-tagged Sjc23-LED protein was purified with the His GraviTrap column (GE Healthcare, Uppsala, Sweden) according to the manual provided by the manufacturer. The protein was eluted using an elution buffer containing 500 mM imidazole. The eluate was analyzed by running on a 12% SDS-PAGE and Western-blot with a mAb to the His-tag. The recombinant proteins of TSP-2 and GST of *S. japonicum* were generated as described [14], [16].

### Binding of Sjc23-LED with four nonimmune human immunoglobulins in ELISA

In an enzyme-linked immunosorbent assay (ELISA), we coated plates with human immunoglobulins (IgG, IgA, IgE and IgM) (250 ng/well) overnight at 4°C.The same amount of human and bovine albumin was used as control proteins. Sjc23-LED were added to the wells in a series of dilutions from 500 µg/ml to 62.5 µg/ml and incubated for 1 h at 37°C. The binding was detected with an ALP-anti-His tag mAb [14].

### Pull-Down assay of Sjc23-LED with four human nonimmune immunoglobulins

The His-tagged Sjc23-LED immobilized on Ni-NTA agarose resin was used as a bait protein, and which was incubated with human non-immune human immunoglobulins, IgG, IgA, IgE and IgM respectively as previously described [29]. Briefly, 100 µg of Sjc23-LED was incubated with 50 µl Ni-NTA Sepharose resin for 30 min at room temperature. After washing to remove unbound protein,100 µg of human IgG, IgA, IgM (Sigma, CA, USA) or IgE (Abcam, Cambridge, UK) was mixed with the Sjc23-LED-bound resin and incubated at 4°C for 1 h with gentle agitation. The GST-bound-glutathione Sepharose resin was used as a control for unspecific binding to the resin by the immunoglobulins. After incubation, the resin was washed five times with 1 ml phosphate buffered saline (PBS) buffer (137 mM NaCl, 2.7 mM KCl, 10 mM sodium phosphate dibasic, and 2 mM potassium phosphate monobasic, pH of 7.4) to remove unbound protein. Proteins were eluted in 100 µl 1 mol/L NaCl by gentle agitation at 4°C for 30 min. Aliquots of the eluted proteins were resolved in 10% SDS-PAGE and blotted to nylon films. The binding was visualized with a polyclonal antibody that recognized all human immunoglobulins.

### Binding of Sjc23-LED to the Fab and Fc fragments of human IgG

Human IgG Fab and Fc fragments (Merck, NJ, USA) were incubated with Sjc23-LED-bound Sepharose resin as described above. The binding was detected in Western-blot with goat anti-human IgG (Fab specific)-ALP and goat anti-human IgG (Fc specific)-ALP (Sigma, CA, USA) respectively.

In ELISA assay, Fab and Fc fragments of human IgG were respectively coated in ELISA plates; Sjc23-LED recombinant protein diluted from 100 to 3.125 µg/ml was incubated with the two antibody fragments. The binding was detected with an anti-His mAb.

### Inhibition of Ig-binding with overlapping synthetic peptides of Sjc23-LED

Seven peptides of 19 amino acids were chemically synthesized to cover the whole sequence of Sjc23-LED [23] with 9–10 amino acids overlapping between adjacent peptides. 100 µg of human IgG was mixed respectively with aliquots of the peptides (from 100 to 10 µg in each reaction) and incubated with the Sjc23-LED-His GraviTrap Sepharose beads at 4°C for 30 min. The beads were washed for 5 times with PBS buffer. The proteins bound to the beads were resolved in 10% SDS-PAGE and blotted to a nylon filter film. The inhibition effect of the peptides on the binding of Sjc23-LED and human IgG was visualized by ALP-conjugated goat anti-human Ig antibodies as described above.

### Analysis of tertiary structure of Sjc23-LED

The amino acid sequence of Sjc23 was aligned with protein sequences in the structural database using the phyre (Protein Homology/analogy Recognition Engine) server at Imperial College (http://www.sbg.bio.ic.ac.uk/phyre/) [30]. The 3-dimensional structure was further illustrated with PyMOL Viewer program (http://www.pymol.org/).

## Supporting Information

Figure S1 **Test of binding of Sjc23 with bovine and human albumin**. Bovine, human albumin proteins and human IgG were coated on ELISA plate and the binding of Sjc23 to these proteins was detected with anti-HIS mAb. The binding to IgG was dilution dependent, while the binding of Sjc23 to both bovine and human albumins was negative.
(TIF)
