# Supplementary figures and images for: Mapping the Binding between the Tetraspanin Molecule (Sjc23) of Schistosoma japonicum and Human Non-Immune IgG
Source: PLoS One. 2011 Apr 20;6(4):e19112. doi: 10.1371/journal.pone.0019112 (PMC3080413; doi:10.1371/journal.pone.0019112)

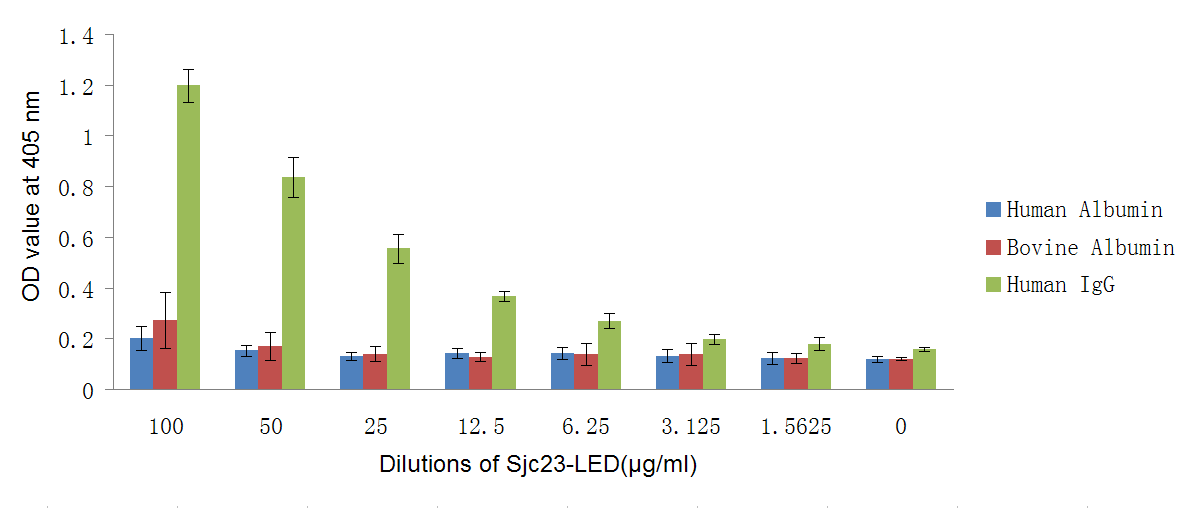

Supplement: Figure S1 — Test of binding of Sjc23 with bovine and human albumin. Bovine, human albumin proteins and human IgG were coated on ELISA plate and the binding of Sjc23 to these proteins was detected with anti-HIS mAb. The binding to IgG was dilution dependent, while the binding of Sjc23 to both bovine and human albumins was negative. (TIF) [file pone.0019112.s001.tif]
